# Serum Levels of BAFF and APRIL Predict Clinical Response in Anti-PLA2R-Positive Primary Membranous Nephropathy

**DOI:** 10.1155/2019/8483650

**Published:** 2019-11-05

**Authors:** Giuseppe Stefano Netti, Barbara Infante, Federica Spadaccino, Giulia Godeas, Maria Grazia Corallo, Concetta Prisciandaro, Laura Croce, Mario Rotondi, Loreto Gesualdo, Giovanni Stallone, Giuseppe Grandaliano, Elena Ranieri

**Affiliations:** ^1^Clinical Pathology Unit and Center for Molecular Medicine, Department of Medical and Surgical Sciences, University of Foggia, Foggia, Italy; ^2^Nephrology Dialysis and Transplantation Unit, Department of Medical and Surgical Sciences, University of Foggia, Foggia, Italy; ^3^Internal Medicine and Endocrinology Unit, Laboratory for Endocrine Disruptors, ICS Maugeri I.R.C.C.S., University of Pavia, Pavia, Italy; ^4^Nephrology Dialysis and Transplantation Unit, Department of Emergency and Organ Transplantation, University of Bari “Aldo Moro”, Bari, Italy; ^5^Fondazione Policlinico Universitario A. Gemelli IRCCS, Rome, Italy; ^6^Università Cattolica del Sacro Cuore, Rome, Italy

## Abstract

Primary membranous nephropathy (PMN) is a renal-specific autoimmune disease caused by circulating autoantibodies that target glomerular podocyte antigens (PLA2R/THSD7A). However, very little is known on the molecular mechanisms controlling B cell response in this nephropathy. The present study was aimed at correlating the serum levels of B cell activators BAFF/BLyS and APRIL with the presence of anti-PLA2R antibodies in PMN patients and with long-term clinical outcome. To this aim, 51 patients with anti-PLA2R-positive biopsy-proven PMN and nephrotic range proteinuria (>3.5 g/24 hours) were enrolled between January 2009 and December 2015 and treated with conventional 6-month immunosuppressive therapy. After 6 months, 29 patients (56.9%) cleared circulating anti-PLA2R, while in remaining 22 (43.1%), they persisted. Intriguingly, in the first group, baseline serum levels of BAFF/BLyS and APRIL were significantly lower than those in the second one. Moreover, after 6 months of immunosuppressive therapy, an overall reduction in both cytokine serum levels was observed. However, in PMN patients with anti-PLA2R clearance, this reduction was more prominent, as compared with those with anti-PLA2R persistence. When related to clinical outcome, lower baseline BAFF/BLyS (<6.05 ng/mL) and APRIL (<4.20 ng/mL) serum levels were associated with significantly higher probability to achieve complete or partial remission after 24-month follow-up. After dividing the entire study cohort into three groups depending on both cytokine baseline serum levels, patients with both BAFF/BLyS and APRIL below the cut-off showed a significantly higher rate of complete or partial remission as compared with patients with only one cytokine above the cut-off, while the composite endpoint was achieved in a very low rate of patients with both cytokines above the cut-off. Taken together, these results provide new insights into the role of BAFF/BLyS and APRIL in both the pathogenesis of anti-PLA2R-positive PMN and the response to immunosuppressive therapy.

## 1. Introduction

Primary membranous nephropathy (PMN) is an immune-mediated glomerular disease caused by circulating autoantibodies targeting glomerular podocytes. The subsequent complement cascade activation along the glomerular basement membrane at the subepithelial level results in a significant injury to the glomerular filtration barrier, leading to proteinuria [1–4]. This nephropathy is the most frequent cause of nephrotic syndrome among primary glomerulonephritis worldwide and represents 20-37% of the cases in most series, rising to 40% in adults over 60 [5–7], while it appears rarely in children (1%-7% of biopsies) [8].

The primary target antigen in this setting is represented by the M-type phospholipase A2 receptor (PLA2R) expressed on the podocyte plasma membrane. Approximately 70-80% of the patients with PMN have detectable serum anti-PLA2R antibodies. Several investigations demonstrated that anti-PLA2R serum levels are strictly associated with disease activity and progression, might be useful to monitor the response to current immunosuppressive therapy, and might reliably predict the chance of recurrence after kidney transplantation [9]. A small number of patients with PMN (approximately 3-5%) have antibodies against thrombospondin type-1 domain-containing 7A (THSD7A) [10, 11], while less than 15-30% of patients are PLA2R/THSD7A negative.

These observations indicate that in the pathogenesis of PMN, humoral immunity may play a pivotal role. However, very little is known on the molecular mechanisms controlling B cell response in this setting.

BAFF (B cell-activating factor belonging to the tumour necrosis factor family) and APRIL (a proliferation-inducing ligand) are members of the tumour necrosis factor (TNF) superfamily. Their main functions are to modulate survival and differentiation of B lymphocytes, although these two soluble mediators present slightly different regulating effects on antibody-mediated immune response [12–14]. BAFF is also called BlyS (B lymphocyte stimulator) or TALL-1 (TNF and apoptosis ligand-related leukocyte-expressed ligand 1). BAFF and APRIL bind to specific receptors on naive or memory B cells and enhance B cell survival.

In addition to these fundamental roles, these cytokines have been suggested to play a pivotal role in promoting B cell shift to self-reactivity [15–17], and elevated blood or tissue levels of BAFF and APRIL are frequently observed in several autoimmune diseases [18, 19] and other B cell-related disorders [20–23]. More recently, the serum levels and the tissue expression of both BAFF and APRIL were analysed in patients with MN and their expression pattern was found to be similar to the one observed in lupus patients [24], but the correlation with anti-PLA2R status was not evaluated.

Herein, we analysed the serum levels of BAFF and APRIL at the time of renal biopsy and after immunosuppressive therapy in patients affected by anti-PLA2R-positive primary MN and investigated their correlation with the main kidney outcomes.

## 2. Methods

### 2.1. Study Design, Patient Cohorts, and Serum Collection

This is a single-centre, retrospective, observational cohort study including 73 patients with biopsy-proven PMN and nephrotic range proteinuria (>3.5 g/24 hours) referred to our institution between January 2009 and December 2015.

All the enrolled patients fulfilled the diagnostic criteria for PMN, as described below. Immune and electron-dense deposits were present exclusively in a subepithelial location, with an absence of deposition within subendothelial or mesangial regions. No glomerular infiltrating cells or proliferation of mesangial and endothelial cells was present. Immunofluorescence demonstrated that IgG was deposited in a granular pattern along the glomerular capillary walls in association with C3. No patients had evidence of secondary features, such as antinuclear antibodies, anti-double-stranded DNA, antinuclear ribonucleoprotein, anti-Smith, anti-Ro (SSA), anti-La (SSB), anti-topoisomerase I (anti-Scl-70), anti-Jo-1 antibodies, anti-cardiolipin antibodies, anti-thyroglobulin antibodies, serum HBV antigens and antibodies, HCV antibodies, HCV RNA, clinical diagnosis of solid neoplasia, or exposure to toxic agents.

Serum samples were collected from all the patients included in the study (*n* = 73) at the time of kidney biopsy. All the patients completed the treatment protocol at our institution, so their serum samples after 6, 12, and 24 months of treatment were also collected. Complete clinical and laboratory data were recorded. All the patients were treated with a 6-month course of intravenous methylprednisolone (1 g intravenously for 3 consecutive days followed by oral methylprednisolone, 0.4 mg/kg per d for 27 days) alternated every other month with oral cyclophosphamide (2.5 mg/kg per day for 30 days) [25].

Serum samples from 12 patients affected by lupus nephritis (III-IV stages according to the ISN/RPS classification) and from 20 healthy volunteers were used as controls. Serum was stored at -80°C before use.

The present study was approved by the local ethical committee (University Hospital “Ospedali Riuniti” Ethical Committee Decision no. 27/CE/2010 of May 10, 2010). All procedures performed in the present study were in accordance with the ethical standards of the last version of the Declaration of Helsinki, and all the enrolled patients provided a written informed consent to participate to the present study.

### 2.2. Anti-PLA2R and Anti-THSD7A Immunoassay

Serum samples of 73 MN patients collected at the time of biopsy and 6 months after therapy were tested for the presence of circulating anti-PLA2R and anti-THSD7A total IgG antibodies with indirect immunofluorescence assay kits (Euroimmun, Lübeck, Germany) following the standard instructions [10, 26]. Patients' plasma was diluted to 1 : 10 and incubated on the reaction fields of slides at room temperature for 30 minutes. After washing, the slides were incubated with FITC-conjugated secondary antibodies at room temperature for 30 minutes. Then, the slides were examined by fluorescence microscopy (Leica Microsystems AG, Wetzlar, Germany).

Antibody positivity was defined as positive staining at serum dilutions of 1/10 or higher. Negativity of anti-PLA2R1 was defined as an absence of detectable antibodies at 1/10 dilution. All samples were assayed in duplicate.

### 2.3. ELISA

The serum level of anti-PLA2R antibodies was also detected quantitatively at baseline and after 6, 12, and 24 months of therapy by the enzyme-linked immunosorbent assay (ELISA) using a commercially available kit (Euroimmun, Lübeck, Germany), following the manufacturer's instructions. The microplate was coated with PLA2R isoform 1. The serum samples were diluted to 1/10 in sample buffer. Anti-PLA2R antibody concentration was determined from the regression line for a standard curve generated using calibration sera with anti-PLA2R at various concentrations (2, 20, 100, 500, and 1500 RU/mL) performed contemporaneously with each assay, as recommended by the manufacturer. If samples were outside the scale of the standard curve, samples were remeasured at a dilution of 1 : 400 or higher. Data were expressed as relative units per milliliter (RU/mL). All samples were assayed in duplicate.

The minimum detectable concentration of anti-PLA2R antibodies was 0.4 RU/mL, while the calculated overall intra-assay coefficient of variation was 3.4%. A value ≥ 14 RU/mL was regarded as a positive result, as suggested by recent studies [27–29].

BAFF/BLyS and APRIL titres in sera of PMN patients were measured by ELISA using commercially available kits (eBioscience, San Diego, CA, USA) following the manufacturer's instructions, as described elsewhere [30]. Both BAFF/BLyS and APRIL concentrations were determined from the regression line for a standard curve generated using highly purified recombinant cytokines at various concentrations performed contemporaneously with each assay, as recommended by the manufacturer's instructions. Data were expressed as ng/mL. The minimum detectable concentration of BAFF/BLyS was 0.13 ng/mL, while the calculated overall intra-assay coefficient of variation was 8.2%. The minimum detectable concentration of APRIL was 0.40 ng/mL, while the calculated overall intra-assay coefficient of variation was 8.1%. All samples were assayed in duplicate.

### 2.4. Outcomes

The primary outcome of this study was the achievement of complete or partial remission [31]. Complete remission (CR) was defined as 24 h proteinuria < 0.5 g in at least two consecutive visits, while partial remission was defined as 24 h proteinuria < 3 g or at least 50% reduction versus baseline values. We considered a composite endpoint (complete or partial remission) defined as the presence of at least one of these two events in the study population.

Patients who did not achieve these outcomes in the study period were considered to have limited response (LR), if a >50% reduction from baseline proteinuria but not achieving ≤3.5 g/d proteinuria was gained, or to be nonresponders (NRs), if a <50% reduction from baseline proteinuria was observed. Patients with progression to end-stage renal disease (ESRD) and the need to switch to another immunosuppressive agent (because of clinical ineffectiveness of the therapeutic protocol) were also considered NRs.

### 2.5. Statistical Analysis

Statistical analysis was performed using SPSS 25.0 software (IBM Corp., Armonk, NY), as previously described [32–36]. Variable distribution was tested using the Kolmogorov-Smirnov test. Continuous variables were compared between groups by Student's *t*-test for unpaired data and Mann-Whitney *U* test, as appropriate. Frequencies were compared among groups by the *χ*^2^ test. A comparison of more than two group means was performed with one-way analysis of variance (ANOVA). Correlation between two variables was ascertained by Pearson or Spearman's correlation tests, as appropriate.

A receiver operating characteristic (ROC) curve analysis was performed to validate the association of both BAFF/BlyS and APRIL baseline serum levels with clinical outcome, and an operational cut-off level was defined to differentiate the likelihood of complete/partial response between the two groups.

The Kaplan-Meier method for censored data was used to analyse the probability of achieving the primary outcomes of complete remission, partial remission, or composite endpoint for PMN patients stratified among different levels of BAFF/BlyS and/or APRIL under or above the cut-off. Survival time was calculated from the beginning of treatment until the date of the event; for the composite endpoint (complete or partial remission), survival time was referred to as the time of partial remission. Patients not achieving remission were considered censored at the time of the last visit. Differences among groups were assessed by the log-rank test.

To test the independent effects of different variables on a patient's survival, univariate and multivariate binary logistic regression analysis was used and partial correlation coefficients were computed and presented as a hazard ratio and 95% confidence intervals (HR; 95% CI). Covariates included in the regression model were baseline levels of serum BAFF/BlyS and APRIL, estimated GFR (using the CKD-EPI formula), and daily proteinuria.

A *p* value < 0.05 was considered statistically significant. Data are reported as mean ± standard deviation (SD) or as percentage frequency, unless otherwise specified.

## 3. Results

We tested the baseline sera from 73 patients with biopsy-proven MN for the presence of autoantibodies reactive with PLA2R using serum diluted to 1 : 10 as previously described [26]. At this serum dilution, 51 of 73 patients (69.9%) presented with serum anti-PLA2R reactivity at the time of renal biopsy, as assessed by the immunofluorescence assay.

The remaining 22 patients were tested for circulating anti-THSD7A antibodies with the indirect immunofluorescence assay, but none were positive. Thus, they were considered double-negative patients.

**Figure 1:**
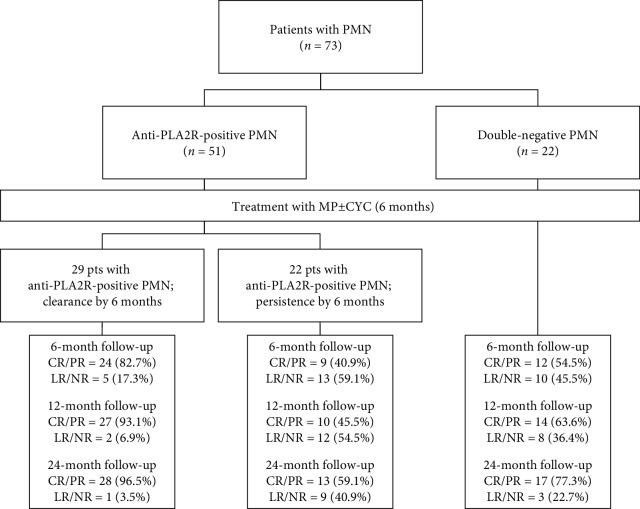
Algorithm of the study. CYC: cyclophosphamide; CR: complete remission; LR: limited response; MP: methylprednisolone; NR: nonresponder; PMN: primary membranous nephropathy; PR: partial remission.

All the PMN patients completed the treatment protocol at our institution: after 6 months of therapy, 29 anti-PLA2R-positive patients became seronegative (group 1) while in 22 anti-PLA2R-positive patients, these autoantibodies were still detectable (group 2). The double-negative patients were also enrolled and treated (group C). The algorithm of the study is described in Figure 1. As summarized in Table 1, the three groups did not differ significantly in the main clinical and laboratory features at the time of renal biopsy.

Noteworthy, patients without anti-PLA2R antibodies after 6 months of therapy (group 1) showed lower baseline anti-PLA2R titre compared with patients with persistent anti-PLA2R antibodies (group 2) (92.3 ± 55.8 vs. 178.2 ± 85.6 RU/mL, *p* = 0.02). Moreover, monitoring of anti-PLA2R serum levels at 6, 12, and 24 months of follow-up in both groups showed a significant reduction of anti-PLA2R titre until disappearance in group 1, while in group 2, an anti-PLA2R persistence, although at slightly lower levels, was observed even at later time points (Table 1). Interestingly, in group 1, a higher rate of complete remission (CR) or partial remission (PR) was observed at 6, 12, and 24 months, as compared to group 2 (Table 1).

**Figure 2:**
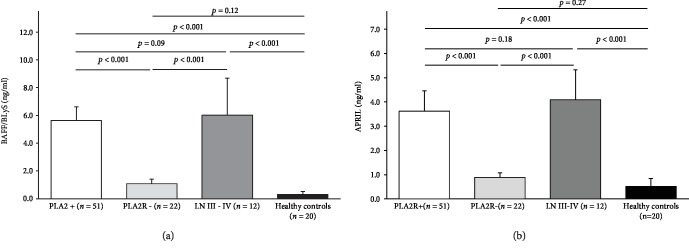
BAFF/BLyS and APRIL baseline serum levels in PMN patients and in controls. (a) BAFF/BLyS baseline serum levels in PMN patients with anti-PLA2R antibodies are significantly higher than those in patients without anti-PLA2R antibodies (5.64 ± 0.97 vs. 1.08 ± 0.33 ng/mL, *p* < 0.001; white and gray histogram, respectively), while no significant difference was observed with those from patients with lupus nephritis (6.01 ± 2.68 ng/mL, *p* = 0.09; dark gray histogram). Finally, in healthy volunteers, BAFF/BLyS was detected at very low concentration (0.31 ± 0.21 ng/mL, *p* = 0.12 vs. PLA2R-negative patients; black histogram). (b) APRIL baseline serum levels in PMN patients with anti-PLA2R antibodies are significantly higher than those in patients without anti-PLA2R antibodies (3.62 ± 0.84 vs. 1.08 ± 0.33 ng/mL, *p* < 0.001; white and gray histogram, respectively), while no significant difference was observed with those from patients with lupus nephritis (4.09 ± 2.24 ng/mL, *p* = 0.18; dark gray histogram). Finally, in healthy volunteers, APRIL was detected at very low concentration (0.52 ± 0.32 ng/mL, *p* = 0.27 vs. PLA2R-negative patients; black histogram). Mann-Whitney *U* test for nonparametric data. Data in the graphs are expressed as mean ± standard deviation.

To elucidate possible B cell-related immune mechanisms underlying the different responses to therapy in our cohort of patients, serum levels of BAFF/BLyS and APRIL were assessed before and after 6 months of therapy in MN patients with anti-PLA2R antibodies at the time of renal biopsy. Moreover, serum samples from 22 patients affected by PMN without anti-PLA2R antibodies, from 12 patients affected by lupus nephritis (III-IV stages according to the ISN/RPS classification), and from 20 healthy volunteers were used as controls. Noteworthy, both BAFF/BLyS and APRIL baseline serum levels in MN patients were comparable with those in patients with lupus nephritis (5.64 ± 0.97 vs. 6.01 ± 2.68 ng/mL for BAFF, *p* = 0.09; 3.62 ± 0.84 vs. 4.09 ± 2.24 ng/mL for APRIL, *p* = 0.18), while these cytokines were detected at very low concentration in MN patients without anti-PLA2R antibodies (1.08 ± 0.33 ng/mL for BAFF, 0.89 ± 0.19 ng/mL for APRIL) and in healthy volunteers (0.31 ± 0.21 ng/mL for BAFF, 0.52 ± 0.32 ng/mL for APRIL) (Figures 2(a) and 2(b)).

In anti-PLA2R-positive MN patients (*n* = 51), serum levels of BAFF/BLyS and APRIL were both reduced after 6 months of therapy (5.64 ± 0.97 vs. 3.26 ± 2.03 ng/mL for BAFF, *p* < 0.001; 3.62 ± 0.84 vs. 1.99 ± 1.63 ng/mL for APRIL, *p* < 0.001). However, no significant change of both cytokines was observed in the double-negative patients after therapy.

**Figure 3:**
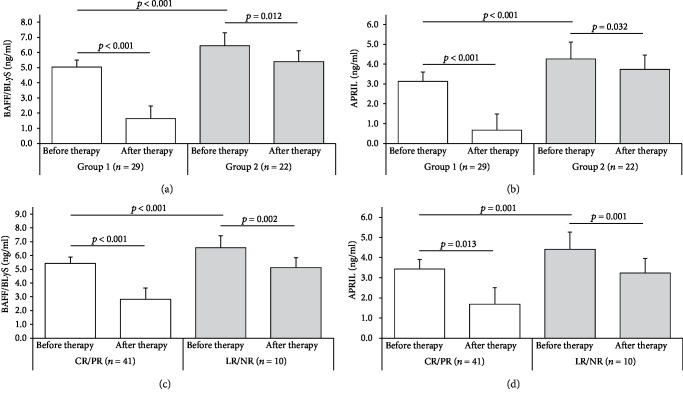
BAFF/BLyS and APRIL serum levels before and after 6-month immunosuppressive therapy among groups. (a) Baseline serum levels of BAFF/BLyS were significantly lower in group 1 (anti-PLA2R clearance after 6-month therapy, *n* = 29) as compared to group 2 (anti-PLA2R persistence after 6-month therapy, *n* = 22) (5.03 ± 0.48 vs. 6.44 ± 0.86 ng/mL, *p* < 0.001). If compared with BAFF/BLyS serum levels after 6-month therapy, really a significant reduction was observed mainly in group 1 (5.03 ± 0.48 vs. 1.64 ± 0.82 ng/mL, *p* < 0.001, white histograms), while in group 2, this reduction, though significant, was less pronounced (6.44 ± 0.86 vs. 5.39 ± 0.73 ng/mL, *p* = 0.012, gray histograms). (b) Baseline serum levels of APRIL were significantly lower in group 1 (anti-PLA2R clearance after 6-month therapy, *n* = 29) as compared to group 2 (anti-PLA2R persistence after 6-month therapy, *n* = 22) (3.12 ± 0.61 vs. 4.26 ± 0.63 ng/mL, *p* = 0.001). If compared with APRIL serum levels after 6-month therapy, really a significant reduction was observed mainly in group 1 (3.12 ± 0.61 vs. 0.67 ± 0.50 ng/mL, *p* < 0.001, white histograms), while in group 2, this reduction, though significant, was less pronounced (4.26 ± 0.63 vs. 3.72 ± 0.63 ng/mL, *p* = 0.032, gray histograms). (c) Baseline serum levels of BAFF/BLyS were significantly lower in PMN patients who achieved complete or partial remission (CR/PR, *n* = 41) after 24-month follow-up, as compared with those with limited response or nonresponders (LR/NR, *n* = 10) (5.41 ± 0.92 vs. 6.56 ± 0.52 ng/mL, *p* < 0.001). Moreover, if the BAFF/BLyS baseline serum levels were compared to those assessed after 6-month follow-up, the cytokine reduction was more marked in patients who achieved the best clinical outcome (CR/PR) after 24-month follow-up (5.41 ± 0.92 vs. 2.81 ± 1.82 ng/mL, *p* < 0.001, white histograms), as compared with the other group (LR/NR) (6.56 ± 0.52 vs. 5.11 ± 1.87 ng/mL, *p* = 0.013, gray histograms). (d) Baseline serum levels of APRIL were significantly lower in PMN patients who achieved complete or partial remission (CR/PR, *n* = 41) after 24-month follow-up, as compared with those with limited response or nonresponders (LR/NR, *n* = 10) (3.43 ± 0.80 vs. 4.41 ± 0.39 ng/mL, *p* = 0.001). Moreover, if the APRIL baseline serum levels were compared to those assessed after 6-month follow-up, the cytokine reduction was more marked in patients who achieved the best clinical outcome (CR/PR) after 24-month follow-up (3.43 ± 0.80 vs. 1.69 ± 1.58 ng/mL, *p* = 0.002, white histograms), as compared with the other group (LR/NR) (4.41 ± 0.39 vs. 3.23 ± 1.25 ng/mL for APRIL, *p* = 0.001, gray histograms). Mann-Whitney *U* test for nonparametric data. Data in the graphs are expressed as mean ± standard deviation.

Baseline serum levels of both BAFF/BLyS and APRIL were significantly lower in group 1 as compared to group 2. After 6 months of therapy, a significant reduction of BAFF/BLyS and APRIL was observed both in group 1 and in group 2, although in the latter the reduction of these cytokines was less pronounced (Figures 3(a) and 3(b)).

We then divided the entire group of MN patients with anti-PLA2R presence at baseline into two groups according to the achievement of complete or partial remission (CR/PR, *n* = 41) or not (LR/NR, *n* = 10) after 24 months follow-up (Figures 3(c) and 3(d)). At baseline, serum levels of both BAFF/BLyS and APRIL were significantly lower in patients who experienced CR/PR after 24 months follow-up as compared with those with LR/NR. Moreover, the cytokine reduction at 6 months after therapy was more marked in patients who achieved the best clinical outcome (CR/PR) after 24-month follow-up as compared with the LR/NR group.

The evolution of serum levels of both BAFF/BLyS and APRIL in response to immunosuppressive therapy during 24-month follow-up suggested a possible role as predictors of anti-PLA2R seroconversion and good clinical outcomes in patients with primary MN (Supplementary [Supplementary-material supplementary-material-1]).

**Figure 4:**
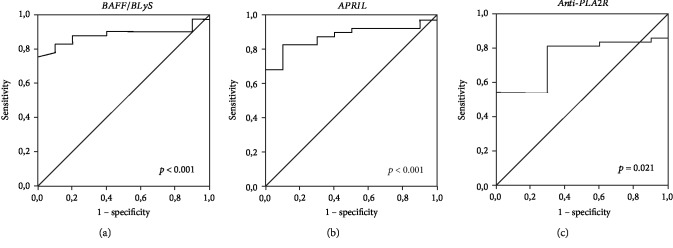
ROC curves for BAFF, APRIL, and anti-PLA2R serum levels and 24-month clinical outcome. (a) ROC curve analysis to validate the association of BAFF/BLyS serum levels at baseline with the probability to obtain CR/PR after 24-month follow-up (AUC = 0.884, CI 95% 0.792-0.976, *p* < 0.001). (b) ROC curve analysis to validate the association of APRIL serum levels at baseline with the probability to obtain CR/PR after 24-month follow-up (AUC = 0.880, CI 95% 0.786-0.975, *p* < 0.001). (c) ROC curve analysis to validate the association of anti-PLA2R serum levels at baseline with the probability to obtain CR/PR after 24-month follow-up (AUC = 0.737, CI 95% 0.598-0.875, *p* = 0.021).

To this aim, a ROC curve analysis was carried out to validate the association of BAFF/BLyS, APRIL, and anti-PLA2R serum levels at baseline with the probability to obtain the composite endpoint of the study (complete or partial remission) after a 24-month follow-up and to define an operational cut-off value. The analysis showed that all three serum parameters were significantly associated with complete or partial remission at 24 months. However, BAFF/BLyS and APRIL serum levels demonstrated to be very good outcome predictors (Figures 4(a) and 4(b)), while anti-PLA2R did not perform well (Figure 4(c)).

**Figure 5:**
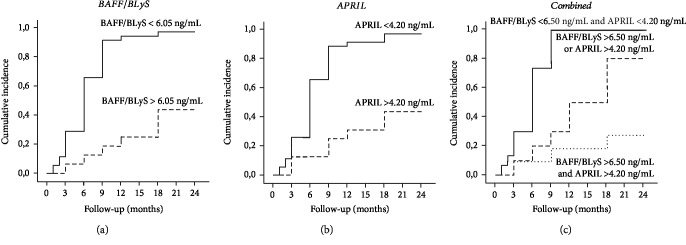
Cumulative incidence of complete/partial remission after immunosuppressive therapy among patients stratified for BAFF and APRIL serum levels. (a) Anti-PLA2R-positive PMN patients with BAFF/BLyS < 6.05 ng/mL at the time of renal biopsy achieved the composite endpoint with a significantly higher percentage, as compared with the patients with BAFF/BLyS above the cut-off (97.1% vs. 43.7%, log-rank test, *p* < 0.001). (b) Anti-PLA2R-positive PMN patients with APRIL < 4.20 ng/mL at the time of renal biopsy achieved the composite endpoint with a significantly higher percentage, as compared with the patients with APRIL above the cut-off (94.4% vs. 46.7%, log-rank test, *p* < 0.001). (c) If the entire study cohort was assigned to three groups depending on both cytokine baseline serum levels, patients with both BAFF/BLyS and APRIL below the cut-off (continuous line) showed a significantly higher complete or partial remission rate as compared with patients with only one cytokine above the cut-off (dashed line) or with patients with both cytokines above the cut-off (dotted line) (100% vs. 80.% vs. 17.3%, Kaplan-Meier lifetime analysis and log-rank test, *p* < 0.001).

For BAFF/BLyS, a cut-off value of 6.05 ng/mL was defined with 90.0% specificity and 82.9% sensitivity and a positive predictive value of 56.2% and a negative predictive value of 97.1%, while for APRIL, a cut-off value of 4.20 ng/mL was defined with 80.0% specificity and 82.9% sensitivity and a positive predictive value of 53.3% and a negative predictive value of 94.4%. We, then, performed a lifetime analysis for the incidence of complete or partial remission after stratification of MN patients according to baseline BAFF/BLyS and APRIL serum levels above or below the cut-off value. An 80.4% overall incidence of complete or partial remission after 24-month follow-up was achieved in the entire study group. Noteworthy, patients with lower BAFF/BLyS and APRIL serum levels at the time of renal biopsy achieved the composite endpoint with a significantly higher percentage, as compared with the patients with higher serum levels of both cytokines (Figures 5(a) and 5(b)). If the entire study cohort was assigned to three groups depending on both cytokine baseline serum levels, patients with both BAFF/BLyS and APRIL below the cut-off showed a significantly higher complete or partial remission rate as compared with patients with only one cytokine above the cut-off, while the composite endpoint was achieved in a very low rate of patients with both cytokines above the cut-off (Figure 5(c)).

Finally, to estimate the relative risk for a good clinical outcome after therapy in anti-PLA2R-positive MN patients, a binary logistic regression analysis was performed using complete or partial remission after 12 months and 24 months as dependent variables and BAFF/BLyS and APRIL serum levels, eGFR, daily proteinuria, and anti-PLA2R serum titre at the time of biopsy as covariates. Intriguingly, while univariate analysis showed that both the B cell cytokines and the anti-PLA2R affected the clinical outcome, in the multivariate analysis, only BAFF/BLyS and APRIL serum levels affected the achievement of the primary outcome, while the other variables did not show any significant association both at 12 and 24 months (Tables 2(a) and 2(b)).

## 4. Discussion

The recent findings on the role of circulating antibodies recognizing podocyte-specific antigens (PLA2R/THSD7A) in the pathogenesis of primary MN have clearly identified this primary glomerulonephritis as an autoimmune disease [36].

Recent studies have shown that approximately 70% to 80% of patients with active primary MN have anti-PLA2R antibodies detected in the serum. Moreover, PLA2R autoantibodies seem to closely correlate with disease activity, and progression can be used to monitor response to immunosuppressive therapy and even predict the risk of recurrence in the renal allograft. However, the systemic immune processes underlying the development of primary MN have not yet been investigated in depth [36].

The present study was aimed at correlating the serum levels of B cell activators BAFF/BLyS and APRIL with the presence of anti-PLA2R antibodies in primary MN patients and with long-term clinical outcome.

In our cohort study, MN patients with anti-PLA2R presence showed significantly higher baseline BAFF/BLyS and APRIL levels, as compared with MN patients negative for anti-PLA2R. As MN patients negative for anti-PLA2R (*n* = 22) resulted also negative for anti-THSD7A antibodies, probably the lower serum levels of B cell cytokines in these patients were consequent to the absence of circulating antibodies, despite the presence of immune deposits in the glomerular basement membrane.

After 6 months of immunosuppressive therapy, among anti-PLA2R-positive patients, 29 (56.9%) cleared circulating anti-PLA2R, while in remaining 22 (43.1%), they persisted. Intriguingly, in the first group, baseline serum levels of BAFF/BLyS and APRIL were significantly lower than those in the second one. Moreover, after 6 months of immunosuppressive therapy, an overall reduction in both cytokine serum levels was observed. However, in primary MN patients with anti-PLA2R clearance, this reduction was more prominent, as compared with those with anti-PLA2R persistence. This data, coupled with the observation of a direct correlation between anti-PLA2R titre and both cytokine serum levels at baseline, suggests a possible pathogenic relationship between anti-PLA2R-positive primary MN and B cell activators.

When related to the clinical outcome, lower baseline BAFF/BLyS and APRIL serum levels were associated with significantly higher probability to achieve complete or partial remission after 24-month follow-up. ROC curve analysis allowed setting a cut-off level of BAFF/BLyS < 6.05 ng/mL and APRIL < 4.20 ng/mL, respectively, which were confirmed as independent factors affecting clinical outcome in both univariate and multivariate binary logistic regressions.

If the entire study cohort was assigned to three groups depending on both cytokine baseline serum levels, patients with both BAFF/BLyS and APRIL below the cut-off showed a significantly higher rate of complete or partial remission rate as compared with patients with only one cytokine above the cut-off, while the composite endpoint was achieved in a very low rate of patients with both cytokines above the cut-off. Taken together, these results provide new insight into the role of BAFF/BLyS in both the pathogenesis of anti-PLA2R-positive PMN and the response to immunosuppressive therapy.

Several reports in humans and animal models suggest that BAFF/BLyS and APRIL may promote autoimmunity. The phenotype of BAFF-transgenic mice resembles the clinical and laboratory features of systemic lupus erythematosus [9]. Also, the overexpression of APRIL in murine models promotes immune dysregulation, although to a lesser degree, through B and T cell activation [37]. Both BAFF/BLyS and APRIL participate in the regulation of B cell survival, immunoglobulin G class switching, and repertoire-selective tolerance [14, 38, 39], supporting the hypothesis that an increase in their production may prime autoreactive B cell clones and induce autoantibody production.

In human studies, increased serum levels of BAFF and APRIL are found in several autoimmune diseases, such as systemic lupus erythematosus (SLE) [40], rheumatoid arthritis [41], and Sjögren's syndrome [42]. The levels of serum BAFF were associated with the presence of autoantibodies in these diseases [43].

A recent report demonstrated the presence of both BAFF/BLyS and APRIL in the renal tissue and the serum of patients with membranous nephropathy, thus correlating their expression trend with lupus patients [24]. Interestingly, B cell activators were detected by both resident and infiltrating cells, thus suggesting an active role in the crosstalk between immune system dysregulation and self-antigen presentation in the pathogenesis of primary MN. However, the possible relationship with anti-PLA2R status was not evaluated.

Our study reports for the first time the possible relationship between both BAFF/BLyS and APRIL serum levels and anti-PLA2R baseline status and seroconversion after therapy in PMN patients. Moreover, lower serum level of both B cell activators predicted good clinical response to immunosuppressive therapy, thus strengthening their potential pathogenic role in this autoimmune nephropathy.

According to the most recent Kidney Disease: Improving Global Outcomes (KDIGO) guidelines, the primary MN first-line immunosuppressive therapy is represented by the “Ponticelli regimen,” a 6-month course of alternating monthly cycles of oral and intravenous steroids and oral chlorambucil/cyclophosphamide [44]. Alternative regimens in patients with specific contraindications to high-dose steroids or alkylating agents are based on the use of calcineurin inhibitors, either cyclosporine or tacrolimus, while corticosteroid monotherapy is not recommended as initial therapy [45]. More recently, several studies have investigated the safety and efficacy of Rituximab, a B cell-depleting agent, in the treatment of patients who have long-lasting proteinuria or who previously failed other treatments. Moreover, Rituximab induces reduction of anti-PLA2R-Ab titre, which seems to precede remission of proteinuria by several months, thus suggesting a causal relationship [27, 46–48]. Despite their reasonable efficiency, better therapeutic strategies are required because the current regimens can have potential side effects after long-term use. In terms of BAFF and APRIL, two different blocking options are currently available for autoimmune diseases and might be evaluated in the setting of primary MN [49, 50].

Obviously, further studies are needed to confirm our data and to evaluate an innovative and tailored therapeutic approach of primary MN, in the attempt to improve the renal outcomes and to reduce the side effects of current treatments.

The major strength of the present study lies on the potential role of baseline BAFF/BLyS and APRIL serum levels as predictive biomarkers of PLA2R seroconversion and long-term good clinical outcome after immunosuppressive therapy in PMN patients. Moreover, our data are of great clinical interest even considering the uniformity of the cohort study and the standardization of the treatment protocol. Nevertheless, our limited sample size requires further studies to confirm our preliminary observations. Moreover, further studies are required to analyse the molecular mechanisms linking B cell activation and PLA2R-negative PMN patients (for instance, anti-THSD7A-positive and double-negative groups).

An incomplete understanding of the pathophysiology of PMN, particularly in terms of the autoimmune system, for a long time has hampered the improvement of clinical outcomes. The present study revealed the possible relationship between both BAFF and APRIL serum levels and anti-PLA2R profile and clinical response to conventional therapy. Nevertheless, more thorough understanding should be obtained by analysing the autoimmune features of PMN, with the aim of improving the clinical management of patients with MN.

## Figures and Tables

**Table 1:** Baseline characteristics and responses to treatment for all subjects grouped according to anti-PLA2R status after 6-month therapy.

	Total	Group 1 (anti-PLA2R positive; clearance at 6 months)	Group 2 (anti-PLA2R positive; persistence at 6 months)	Group 3 (anti-PLA2R negative)	*p*
Baseline characteristics					(Among groups)
Number	73	29	22	22	
Age (years)	53.0 ± 16.6	49.8 ± 19.9	60.2 ± 12.0	50.1 ± 14.1	0.089^∗^
Gender (M/F)	49/24	24/5	13/9	12/10	0.142
Serum creatinine (mg/dL)	1.13 ± 0.64	1.20 ± 0.77	1.12 ± 0.56	1.04 ± 0.55	0.891^#^
Creatinine clearance (mL/min per 1.73 m^2^)	81.9 ± 34.9	87.3 ± 40.7	73.0 ± 25.6	83.8 ± 34.6	0.183^∗^
Serum albumin (g/dL)	2.3 ± 0.9	2.4 ± 0.8	2.2 ± 1.1	3.1 ± 1.0	0.893^∗^
Baseline proteinuria (g/day)	6.8 ± 4.9	8.2 ± 4.3	8.9 ± 5.3	5.7 ± 2.0	0.804^∗^
Responses to treatment					(Group 1 vs. 2)
Baseline anti-PLA2R (RU/mL)	129.4 ± 81.7	92.3 ± 55.8	178.2 ± 85.6	—	<**0.001**^∗^
6-month anti-PLA2R (RU/mL)	70.0 ± 59.0	27.9 ± 22.4	125.6 ± 43.8	—	<**0.001**^∗^
12-month anti-PLA2R (RU/mL)	55.3 ± 54.1	14.6 ± 1.9	108.9 ± 40.9	—	<0.001^∗^
24-month anti-PLA2R (RU/mL)	51.6 ± 54.4	11.6 ± 1.3	104.4 ± 43.6	—	<0.001^∗^
6-month outcome (CR/PR vs. LR/NR)	45 vs. 28	**24** vs. **5**	**9** vs. **13**	12 vs. 10	**0.005**
12-month outcome (CR/PR vs. LR/NR)	51 vs. 22	**27** vs. **2**	**10** vs. **12**	14 vs. 8	<**0.001**
24-month outcome (CR/PR vs. LR/NR)	58 vs. 13	**28** vs. **1**	**13** vs. **9**	17 vs. 3	<**0.001**

Summary data are presented as means ± standard deviation. M: male; F: female; CR: complete remission; LR: limited response; NR: nonresponder; PR: partial remission. ^#^Normal distribution; ^∗^nonnormal distribution.

**(a):** 

	Univariate analysis				Multivariate analysis			
		CI 95%				CI 95%		
	HR	Lower	Higher	*p* value	HR	Lower	Higher	*p* value
BAFF/BLyS (>6.1 ng/mL)	0.177	0.075	0.420	<**0.001**	0.150	0.046	0.485	**0.002**
APRIL (>4.2 ng/mL)	0.291	0.131	0.646	**0.002**	0.331	0.130	0.842	**0.020**
eGFR (mL/min)	1.006	0.997	1.015	0.203	1.000	0.992	1.009	0.917
Daily proteinuria (g/day)	0.949	0.889	1.013	0.119	0.949	0.856	1.052	0.318
Anti-PLA2R (RU/mL)	0.993	0.989	0.998	**0.006**	1.003	0.994	1.011	0.519

**(b):** 

	Univariate analysis				Multivariate analysis			
		CI 95%				CI 95%		
	HR	Lower	Higher	*p* value	HR	Lower	Higher	*p* value
BAFF/BLyS (>6.1 ng/mL)	0.181	0.076	0.435	<**0.001**	0.157	0.047	0.525	**0.003**
APRIL (>4.2 ng/mL)	0.256	0.110	0.594	**0.001**	0.328	0.125	0.855	**0.023**
eGFR (mL/min)	1.005	0.996	1.014	0.277	1.000	.991	1.009	0.974
Daily proteinuria (g/day)	0.941	0.879	1.007	0.079	0.940	0.846	1.044	0.250
Anti-PLA2R (RU/mL)	0.993	0.988	0.998	**0.005**	1.003	0.994	1.011	0.547

CI: confidence interval; HR: hazard ratio; eGFR: estimated glomerular filtration rate.

## Data Availability

All the data used to support the findings of this study are available from the corresponding author upon request.

## Abbreviations

CR: Complete remission
CYC: Cyclophosphamide
eGFR: Estimated glomerular filtration rate
ESRD: End-stage renal disease
LR: Limited response
MN: Membranous nephropathy
NR: Nonresponder
PMN: Primary membranous nephropathy
PR: Partial remission.

## Supplementary Materials

Supplementary Materials Representative evolution of BAFF/BLyS, APRIL, anti-PLA2R, and proteinuria between groups of anti-PLA2R-positive PMN patients (group 1: PMN patients who cleared anti-PLA2R after 6-month therapy; group 2: PMN patients with persistent anti-PLA2R after 6-month therapy) during 24-month follow-up. In the graphs, 5 representative cases per group are shown.
