# Supplementary material for: Serum Levels of BAFF and APRIL Predict Clinical Response in Anti-PLA2R-Positive Primary Membranous Nephropathy
Source: J Immunol Res. 2019 Nov 5;2019:8483650. doi: 10.1155/2019/8483650 (PMC6874868; doi:10.1155/2019/8483650)
Supplement: Supplementary Materials — Representative evolution of BAFF/BLyS, APRIL, anti-PLA2R, and proteinuria between groups of anti-PLA2R-positive PMN patients (group 1: PMN patients who cleared anti-PLA2R after 6-month therapy; group 2: PMN patients with persistent anti-PLA2R after 6-month therapy) during 24-month follow-up. In the graphs, 5 representative cases per group are shown. [file 8483650.f1.docx]

### Supplementary Figure 1. BAFF/BLyS, APRIL, anti-PLA2R and proteinuria between groups of PMN patients during 24-months follow-up


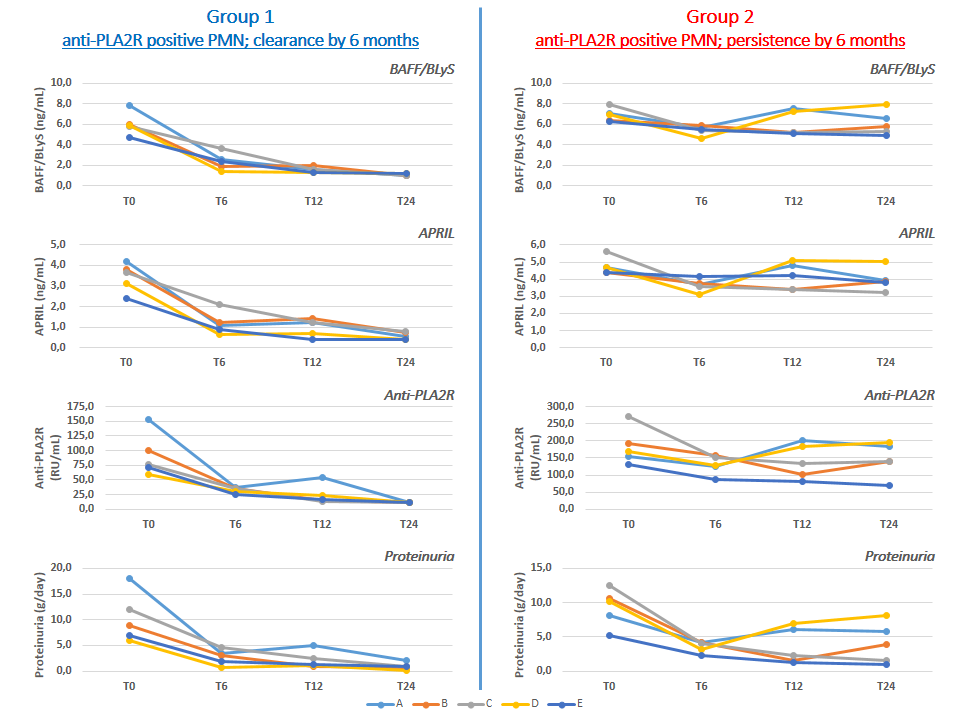


Representative evolution of BAFF/BLyS, APRIL, anti-PLA2R and proteinuria between groups of anti-PLA2R positive PMN patients (Group 1: PMN patients who cleared anti-PLA2R after 6 months therapy; Group 2: PMN patients with persistent anti-PLA2R after 6 months therapy) during 24-months follow-up. In the graphics 5 representative cases per group are shown.
